# Management and Medical Care for Individuals with Type 1 Diabetes Running a Marathon

**DOI:** 10.3390/jcm14072493

**Published:** 2025-04-06

**Authors:** Michał Kulecki, Marcin Daroszewski, Paulina Birula, Anita Bonikowska, Anna Kreczmer, Monika Pietrzak, Anna Adamska, Magdalena Michalak, Alicja Sroczyńska, Mateusz Michalski, Dorota Zozulińska-Ziółkiewicz, Andrzej Gawrecki

**Affiliations:** 1 Department of Internal Medicine and Diabetology, Poznan University of Medical Sciences, Mickiewicza 2, 60-834 Poznań, Poland; paulina.birula@gmail.com (P.B.); kaczmarek.anitaa@gmail.com (A.B.); aniakreczmer7@gmail.com (A.K.); aadamska@ump.edu.pl (A.A.); magdalenamichalak@ump.edu.pl (M.M.); mmichalski@ump.edu.pl (M.M.); dzozulinskaziolkiewicz@ump.edu.pl (D.Z.-Z.); andrzej.gawrecki@ump.edu.pl (A.G.); 2 Doctoral School, Poznan University of Medical Sciences, 61-701 Poznań, Poland; 3 University Centre for Sports and Medical Studies, Poznan University of Medical Sciences, Rokietnicka 5E, 60-806 Poznań, Poland; marcin.daroszewski@gmail.com; 4 Department of Diabetology and Internal Medicine, Raszeja City Hospital Poznań, Mickiewicza 2, 60-834 Poznań, Poland; przybylekmonika88@gmail.com (M.P.); sroczynska.alicja@gmail.com (A.S.)

**Keywords:** type 1 diabetes, physical activity, physical endurance, continuous glucose monitoring, hypoglycemia, marathon

## Abstract

**Background**: Limited data exist on managing type 1 diabetes mellitus (T1DM) during long-distance endurance events such as marathons. This study aimed to assess glycemic control and participant safety during a marathon. **Methods**: Five men with T1DM, participating in the 22nd Poznan Marathon, were recruited. They completed health questionnaires and received training on glycemic management. Their physical capacity was assessed (including maximal oxygen uptake on a cycle ergometer). Participants reduced their insulin doses and consumed breakfast 2.5–3 h before the race. During the marathon, self-monitoring blood glucose (SMBG) and ketone levels were measured at five checkpoints (start, 10 km, 19 km, 30 km, and finish). The medical team followed a pre-approved protocol, providing carbohydrate and fluid supplementation as needed. Glycemia was monitored by two continuous glucose monitoring (CGM) systems (FreeStyle Libre 2 and Dexcom G6) and SMBG. **Results**: The participants’ median age was 44 years (34–48), with a diabetes duration of 10 years (6–14), and a BMI of 22.5 kg/m^2^ (22.0–23.3). All finished the marathon in an average time of 4:02:56 (±00:43:11). Mean SMBG was 125.6 (±43.5) mg/dL, while CGM readings were 149.6 (±17.9) mg/dL (FreeStyle Libre 2) and 155.4 (±12.9) mg/dL (Dexcom G6). One participant experienced prolonged hypoglycemia undetected by CGM, whereas another developed symptomatic hypoglycemia between SMBG measurements. **Conclusions**: Safe marathon completion in people with T1DM requires individualized insulin dose adjustments, appropriate carbohydrate supplementation, and dedicated medical support at checkpoints. Combining CGM with periodic SMBG measurements further enhances safety and helps to detect potential glycemic excursions.

## 1. Introduction

Physical activity significantly benefits individuals with type 1 diabetes (T1DM), similar to the general population. These advantages include an improved insulin sensitivity, lipid profile, bone health, psychological well-being, and reductions in cardiovascular risk, body weight, and blood pressure [1].

Type 1 diabetes results from the autoimmune destruction of pancreatic beta cells, leading to insulin deficiency [2]. Managing T1DM requires balancing insulin administration and carbohydrate intake, especially during physical activity. According to a Position Statement of the American Diabetes Association, glycemic responses to exercise are highly variable and depend on factors such as activity type, timing, intensity, and circulating insulin levels. To maintain glycemic stability during and after exercise, personalized adjustments in insulin dosing and additional carbohydrate intake are often necessary, supported by frequent blood glucose monitoring. Continuous glucose monitoring (CGM) can aid in detecting trends and preventing hypoglycemia, though it is best used as an adjunct to capillary glucose testing during physical activity [3].

Engaging in high-intensity or prolonged physical activity presents unique glycemic challenges, including exercise-induced hypoglycemia and insulin adjustments in response to fluctuating energy demands [4]. Despite many guidelines dedicated to physical activity in diabetes [5,6,7], as well as more specific recommendations for athletes [8], there is still insufficient scientific evidence for long-distance running.

Recent advances in diabetes treatment have enabled individuals with type 1 diabetes to participate in professional sports competitions, including the Olympic Games, World Championships, and European Championships [9,10]. Studies have documented successful marathon completions by athletes with T1DM, achieved through careful planning, carbohydrate adjustments, and close blood sugar monitoring [11,12]. During prolonged and intense physical activities, such as marathons, the body’s increased glucose utilization and enhanced insulin sensitivity can cause hypoglycemia. This challenges T1DM long-distance runners and their healthcare teams [1]. The fear of hypoglycemia remains the strongest barrier to physical activity [13]. Despite this concern, more people with T1DM are participating in endurance events such as marathons [10].

Marathons, spanning 42.195 km, are a demanding form of long-distance aerobic exercise, even for individuals without diabetes.

Continuous glucose monitoring (CGM) systems improve diabetes management by offering real-time data on interstitial glucose levels [14]. CGM systems help athletes with T1DM to maintain stable glucose levels during extended activities like marathons [15]. Most research focuses on shorter endurance events such as half-marathons [16,17].

While strategies exist for managing glycemia during exercise in T1DM athletes, knowledge about marathon running in T1DM amateurs is still limited and requires further research [5].

Therefore, this study aims to assess glycemic control and participant safety during a marathon, specifically evaluating self-monitoring blood glucose (SMBG) measurements and continuous glucose monitoring (CGM) in adults with T1DM.

## 2. Materials and Methods

We included five male participants with T1DM who had completed a marathon. Written informed consent was obtained from all participants. This study adhered to the ethical guidelines set by the local Ethical Committee (approval No.1245/18, 6 December 2018) and followed the principles of the Declaration of Helsinki [18]. Participants with confirmed T1DM were recruited between August and September 2023 through flyers, online advertisements, and collaborations with local marathon organizations.

### 2.1. Inclusion and Exclusion Criteria

The inclusion criteria were as follows:

- A diagnosis of T1DM for at least 1 year;
- Age ≥ 18 years;
- Written informed consent and adherence to the study protocol.

The exclusion criteria included the following:

- Pregnancy;
- Use of medications significantly affecting metabolism or exercise performance;
- The presence of chronic medical or psychiatric conditions limiting safe participation;
- The presence of advanced chronic complications of diabetes: proliferative retinopathy, diabetic kidney disease in stages III–V, cardiovascular diseases;
- The inability to safely complete maximal exercise testing.

### 2.2. Baseline Assessment

Each participant filled out a form covering their medical history, complications, coexisting conditions, and smoking habits. Initial evaluations involved reviewing runnerhistory and a physical examination.

A thorough baseline assessment took place on one visit, gathering data on demographics, medical background, and diabetes management practices. Demographic details included age, sex, height, weight, and BMI.

The medical history section included T1DM duration, any diabetes-related complications, and other significant health issues. Details on insulin treatment type—either multiple daily injections (MDI) or continuous subcutaneous insulin infusion (CSII)—daily insulin dose, and HbA1c levels (using a standardized test) were documented.

Participants also reported their blood glucose monitoring frequency, any history of hypoglycemia, and regular physical activity habits, specifying the type, duration, and frequency of exercise over the previous year.

### 2.3. Pre-Race Preparations

All participants underwent training on exercise-related glycemic control. They were advised to reduce their pre-prandial insulin dose and consume breakfast 2.5–3 h before the marathon. The individuals were equipped with 2 CGM systems—FreeStyle Libre 2 (FSL2) (five participants) and Dexcom G6 (DG6) (four participants) under standard, insurer-reimbursed care. They were instructed on the proper use of these devices. The alarms for hyperglycemia and hypoglycemia were turned on. The sensors started at least 24 h before the marathon.

### 2.4. Marathon Monitoring and Support

We designed the protocol to maintain stable glycemia and ensure participant safety by integrating real-time blood glucose monitoring with structured checkpoint assessments based on the EASD/ISPAD/ADA 2020 recommendations and a publication describing glycemic management at the Paris Marathon [5,12]. Before the race, each runner aimed to achieve a recommended glycemic range, and carbohydrate intake or insulin dose adjustments were guided by threshold-based indications. Measurements of blood glucose and ketones were taken at specified intervals during the marathon, and any concerning results triggered targeted interventions, such as partial insulin correction to mitigate the risk of excessive hypoglycemia. If ketone levels rose to a point suggesting potential progression to ketoacidosis, runners were instructed to discontinue the race for their safety. The medical team, monitoring real-time data via a cloud-based platform, provided individualized support and recalibrated runners’ management plans as needed. Table 1 details these specific thresholds and recommendations, illustrating carbohydrate supplementation and insulin adjustment according to current glycemia.

During the marathon, five checkpoints were placed along the course (start, 10 km, 19 km, 30 km, and finish) to facilitate SMBG and ketone level measurements. At each checkpoint, participants briefly stopped to undergo an assessment by the medical team. The SMBG was measured using the Contour Plus One glucometer (Ascensia Diabetes Care, Basel, Switzerland) while ketone levels were measured using the Optium Xido (Abbott Diabetes Care, Alameda, CA, USA). The runner’s finger was disinfected and dried, and then the test strips were checked and validated for accuracy before use. Concurrently, another medical team member measured the capillary ketonemia using the designated device, following the manufacturer’s guidelines.

We also provided participants with isotonic fluids and simple carbohydrates at each checkpoint. If participants’ blood glucose levels fell outside the established range, they were given additional carbohydrates or required insulin adjustments. Each participant was also equipped with glucagon for emergency treatment in case of severe hypoglycemia.

Throughout the marathon, SMBG and CGM data were collected at each checkpoint. These data were analyzed to assess glucose trends and the effectiveness of glycemic control strategies implemented during the marathon. Participants’ feedback on their experiences and any hypoglycemic or hyperglycemic events were also documented.

### 2.5. Cardiopulmonary Exercise Testing (CPET)

We used a metabolic cart (e.g., Vyntus CPX, Vyaire Medical, Mettawa, IL, USA) during a maximal exercise test on a specialized cycle ergometer (e.g., Excalibur Sport 2, Lode, Groningen, The Netherlands) to measure peak oxygen uptake (VO_2max_). The system was calibrated before each test following the manufacturer’s guidelines.

The cardiopulmonary exercise testing (CPET) protocol began with baseline gas exchange measurements while participants rested comfortably. A three-minute warm-up of light cycling followed this. The exercise phase started with a low workload, increasing progressively by 15–20 watts per minute until participants reached volitional exhaustion or chose to stop due to symptoms.

### 2.6. Data Analysis

We summarized the participants’ characteristics, physical capacity, and metabolic parameters using descriptive statistics (median and interquartile range). The maximal predicted oxygen uptake for men was calculated following the Wasserman and Hansen method [19,20]:

W = (predicted VO_2max_ − VO_2unloaded_)/103

Glucose data were obtained from continuous glucose monitoring (CGM) systems and visualized using Microsoft Excel for Microsoft 365, Version 2402 (Build 17328.20142) (Microsoft Corporation, Redmond, WA, USA). Data were exported from two CGM data platforms: Dexcom Clarity (Dexcom Inc., San Diego, CA, USA) and LibreView (Abbott Diabetes Care, Alameda, CA, USA) [21,22]. These platforms provided timestamped interstitial glucose readings, which were used to generate daily glucose trend graphs and assess glycemic variability during exercise.

To assess the accuracy of the two CGM systems, each system’s performance was compared to SMBG measurements as the reference method. The Mean Absolute Relative Difference (MARD) was calculated for each device using paired data points from the CGM systems and the corresponding glucometer readings [23].

The normality of the MARDs was verified using the Shapiro–Wilk test. Subsequently, a paired *t*-test was performed to compare the MARD values between DG6 and FSL2.

## 3. Results

All participants were men, with a median age of 44 years (34–48) and a median diabetes duration of 10 years (6–14). Table 2 provides an overview of their basic characteristics. They maintained good glycemic control, with a median HbA1c of 5.8% (5.6–6.9%). They had a normal median BMI of 22.5 kg/m^2^ (22.0–23.3). Each participant had at least five years of running experience, and three had previously completed a marathon. All participants showed hypoglycemia awareness, as assessed by the Clarke hypoglycemia questionnaire. None had severe hypoglycemia or ketoacidosis in the year before the marathon.

Three out of five participants achieved a time in range (TIR) of over 70% in the 90 days before the marathon, reflecting good pre-competition glycemic control. Glycemic variability showed a coefficient of 34.6% (27.8–39.5), indicating moderate variability.

All participants received multiple daily injections of insulin analogs; none used an insulin pump. The median basal insulin dose was 13 units (7–22), and the median prandial dose was 16.5 units (12–24). Participant 3 did not use basal insulin, whereas the remaining participants used ultralong-acting insulin analogs; they did not reduce their doses before the marathon. Daily insulin intake ranged from 0.26 to 0.57 units/kg body weight.

Participants reported no severe coexisting conditions. One had hypothyroidism (with thyroid-stimulating hormone within the normal range) and hyperlipidemia, and the other one was treated due to hypertension. The others had no health issues aside from T1DM. Their VO_2max_ reached a median of 44.2 mL·kg^−1^·min^−1^ (36.5–44.3 mL·kg^−1^·min^−1^).

All participants completed the marathon safely, although three experienced symptomatic hypoglycemia despite interventions. The maximum ketonemia during the marathon was 0.3 mmol/L (1.74 mg/dL).

**Figure 1:**
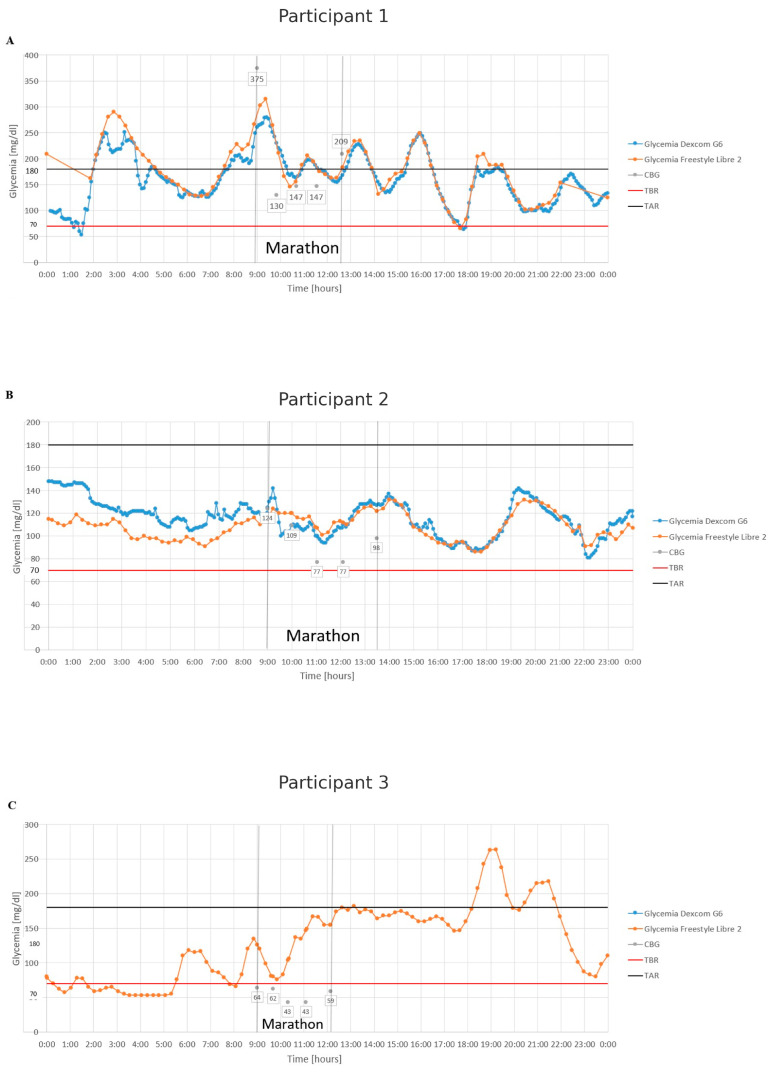
Glycemia of runners with type 1 diabetes on the day of the marathon. Abbreviations: CBG, capillary blood glucose; TBR, time below range; TAR, time above range. (**A**) Participant 1. (**B**) Participant 2. (**C**) Participant 3.

The first participant, a 55-year-old marathon novice, faced hyperglycemia at the race’s start. After administering 2 units of rapid-acting insulin, he finished the marathon in 3 h and 38 min without hypoglycemia (Figure 1A).

The second participant, aged 38, maintained 100% time in range (TIR) due to a partial remission period, achieving excellent metabolic control. He completed the marathon in 4 h and 28 min, keeping his glycemia stable. Notably, his capillary blood glucose levels at 19 km, 30 km, and the finish were lower than the readings from CGM (Figure 1B).

The third participant, the youngest in the group, had the highest VO_2max_ and the fastest marathon time of 3 h and 8 min. Starting the run with hypoglycemia, he consistently consumed carbohydrates but still experienced hypoglycemic symptoms, particularly near the finish, which slowed him down. His glucometer readings also differed from the FSL2 sensor. Dexcom G6 data were unavailable due to an incompatible phone (Figure 1C).

**Figure 2:**
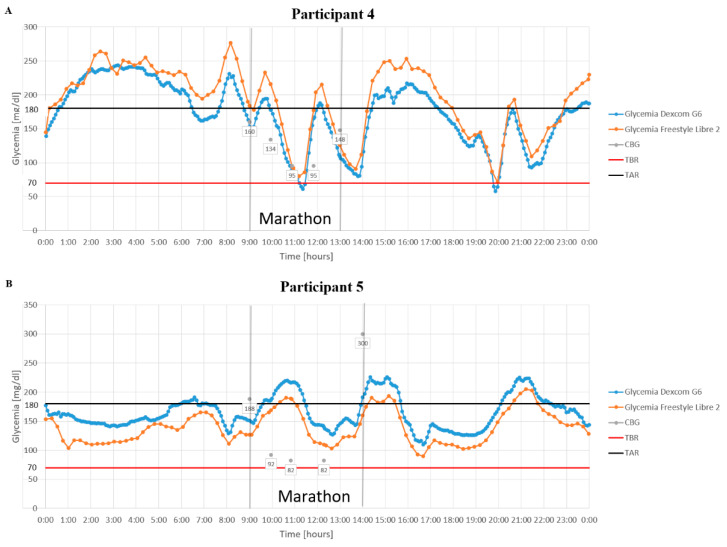
Glycemia of runners with type 1 diabetes on the day of the marathon. Abbreviations: CBG, capillary blood glucose; TBR, time below range; TAR, time above range. (**A**) Participant 4. (**B**) Participant 5.

The fourth participant, aged 34, experienced symptomatic hypoglycemia between the 19 km and 30 km marks. This required him to pause and consume carbohydrates. His Dexcom G6 detected hypoglycemia, but the capillary tests missed it since the episodes occurred between monitoring points (Figure 2A).

**Figure 3:**
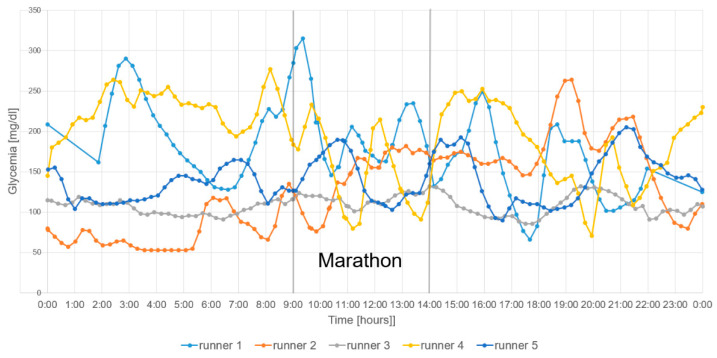
Glycemia of all participants.

The fifth participant and marathon novice, aged 44, finished the marathon in just under 5 h, the slowest time. He had the lowest VO_2max_. He avoided hypoglycemia, though his capillary glucose readings were lower than CGM data. After consuming carbohydrates at 30 km, he developed hyperglycemia, with his capillary blood glucose exceeding 300 mg/dL. He required an insulin bolus at the finish line (Figure 2B). Figure 3 shows the glycemia profiles of all participants.

The mean MARD for Dexcom was 40.2% (±40.2), while for FreeStyle Libre it was 38.1% (±32.9). A paired *t*-test showed no statistically significant difference between the two systems (mean difference = 0.02, *t*(19) = 0.58, *p* = 0.57).

## 4. Discussion

The participants demonstrated good pre-race glycemic control, showing that well-managed T1DM can support effective training for endurance events. Despite this, three participants experienced symptomatic hypoglycemia during the race, even with continuous glucose monitoring (CGM) and capillary blood glucose (SMBG) testing. This highlights the ongoing challenge of maintaining stable glucose during prolonged aerobic exercise. Discrepancies between the CGM and SMBG readings further emphasized the need for multiple monitoring methods to manage rapid glucose fluctuations effectively.

### 4.1. Marathon with Type 1 Diabetes

The first documented case of a man with T1DM completing a marathon appeared in 1987 in the British Journal of Sports Medicine. This 35-year-old man had managed T1DM for 17 years without complications [24]. In 1992, the International Diabetic Athletes Association supported 13 runners with T1DM during the New York City Marathon, demonstrating that strategic planning can enable T1DM athletes to participate safely in endurance sports [11].

In another study, Thuilier et al. observed 12 participants with T1DM running the Paris Marathon using CGM. Based on their findings, they proposed a strategy to maintain stable glycemia levels throughout the marathon. At the start of each race, they targeted an initial glycemic range of 140 to 200 mg/dL, with participants having breakfast three hours before the race and undergoing a pre-race blood glucose check. Carbohydrate intake or insulin boluses were adjusted based on pre-race glucose levels, and ketone concentrations were measured pre- and post-race. With checkpoints at the 19th, 30th, and 42nd kilometers, all participants safely completed the marathon without hypoglycemia episodes [12]. Compared to the runners in the 2016 Paris Marathon, our participants were older (44 vs. 40 years), had lower HbA1c (5.8 vs. 6.8%), had a longer diabetes duration (10 vs. 8 years), and had a slightly lower BMI (22.5 vs. 23.1 kg/m^2^).

Another study described the achievements of four men with type 1 diabetes mellitus (T1DM) who participated in an 82 km mountain ultramarathon. All participants managed their diabetes using continuous subcutaneous insulin infusion [25]. Weiss et al. reported the case of a man with T1DM using CGM and an insulin pump who, at the age of 66, completed his sixth 6-day ultramarathon, finishing third overall with a total distance of 467.424 km [26]. The findings demonstrated that individuals with T1DM can successfully undertake extreme physical challenges. Medical care and support during extreme fatigue is advisable and increases the safety of participants. In our study, this was valuable, especially for the two debutants (participants 1 and 5).

### 4.2. Glycemia During the Marathon

Despite thorough pre-race preparation, three of our T1DM participants experienced symptomatic hypoglycemia during the marathon. These individuals had lower glycemia at the beginning compared to those without hypoglycemia episodes during the race. Prasanna et al. showed that in T1DM individuals, pre-exercise glucose values were a strong predictor of hypoglycemia risk, emphasizing the importance of initial glucose levels in reducing the hypoglycemia incidence during physical activity [27].

In contrast, studies performed in healthy individuals, such as the one by Hiromatsu et al., found that varying carbohydrate intake did not significantly impact interstitial glucose levels during exercise, suggesting that carbohydrate management is more straightforward in those without T1DM [28]. Marathon running, a high-energy, long-distance aerobic activity, relies heavily on muscle and liver glycogen as primary energy sources [10]. In healthy individuals, insulin secretion naturally decreases during endurance exercise due to increased sympathoadrenal activity, with adjustments based on exercise intensity and blood glucose levels [9,10]. However, in T1DM, the absence of endogenous insulin secretion complicates glucose regulation, increasing the risk of both hypoglycemia and hyperglycemia [9,10]. Consequently, T1DM runners typically spend less time within the euglycemic range (70–180 mg/dL) than healthy individuals [29]. Despite this, runners with T1DM and at least five years of training often achieve half-marathon times comparable to their healthy peers [17].

### 4.3. Continuous Glucose Monitoring

Participants in our study maintained an average HbA1c of 5.8%, suggesting effective long-term glycemic control. Three out of five participants achieved over 70% time in range (TIR) for the 90 days before the marathon, reflecting good pre-race glycemic management. Although their moderate glycemic variability coefficient did not appear to impact marathon performance, it may have related to hypoglycemia episodes during the marathon [2,10].

Studies have highlighted the value of advanced tools like CGMs in managing blood glucose during exercise [5,30,31]. Case studies confirm that T1DM runners can complete marathons with CGM support and strategic carbohydrate intake [12,32]. Van Weenen et al. analyzed CGM data from professional cyclists with type 1 diabetes, demonstrating that CGM use during training and recovery helped to visualize glycemic trends and supported real-time adjustments to insulin and nutrition strategies. This facilitated improved glucose management across the varying physical demands of elite competition [33]. Adolfsson et al. evaluated endurance-trained athletes with T1DM and found that CGM-supported carbohydrate strategies during prolonged exercise enhanced glucose stability and reduced the risk of hypoglycemia, emphasizing the role of CGM in sustaining safe performance during extended physical effort [34]. Iscoe et al. used CGM in a sports camp setting and highlighted the frequent occurrence of nocturnal hypoglycemia in active individuals with type 1 diabetes. Their findings supported CGM as an essential safety tool for managing glucose fluctuations in athletes following intense daytime training [35].

Our study suggests that using both CGM and SMBG testing improves the probability of detecting hypoglycemia and hyperglycemia during a marathon, as both methods provide complementary data points for glucose regulation.

CGM technology played a crucial role for our participants in monitoring glucose fluctuations during the race, though differences between SMBG and CGM readings were noted. The MARD values for both CGM systems were relatively high, and the corresponding standard deviations indicated substantial variability in measurement accuracy. Previous studies have shown that CGM accuracy decreases during physical activity, particularly throughout periods of rapid glucose change [36]. The differences between CGM and capillary readings were noted by Gamarra et al. in T1DM individuals during scuba diving [37]. Depending on the CGM device, the overall mean of all MARDs during different types of exercise in people with type 1 diabetes is approximately 13.63% (95% CI 11.41%, 15.84%). The time lag between blood glucose and sensor glucose also rises—from around 5 min at rest to 12–24 min during physical activity. Notably, the faster the rate of glucose change, the greater the lag between blood and sensor glucose values [5]. Moser et al. found that CGM showed a MARD ranging from 12.8% to 26.5% during exercise, with the highest in moderate-intensity exercise. Although CGM readings were significantly correlated with capillary glucose (r = 0.74–0.99), they tended to overestimate glucose values during high-intensity efforts. [38]. CGMs with predictive hypoglycemia alerts may improve safety during endurance events by allowing for proactive interventions [39].

### 4.4. Insulin Pumps

Insulin pumps offer greater flexibility for adjusting basal rates compared to injection regimens. As a result, basal dose adjustments concern people using pumps, particularly during unplanned exercise. In contrast, bolus dose adjustments are applicable across most insulin regimens [40]. The users of hybrid closed-loop insulin pumps are recommended to undergo pre-emptive basal insulin reduction or exercise modes with higher glycemic targets initiated 90 min (1–2 h) before the start of exercise until the end of exercise [41]. Based on the EASD/ISPAD position statement, insulin pump therapy, particularly when integrated with automated insulin delivery (AID) systems, offers enhanced flexibility for glucose management during prolonged endurance activities such as long-distance running. AID features, like adjustable glucose targets and temporary insulin delivery modulation, can help to mitigate the risk of hypoglycemia when activated 1–2 h before physical activity. However, real-time carbohydrate intake, continuous glucose monitoring, and individualized strategies remain essential to safely manage glucose fluctuations during extreme exertion [42].

Insulin pump use during a marathon may pose challenges. Physical activity, sweat, and prolonged movement can increase the risk of device dislodgement, infusion site failure, or occlusion. The need to carry and monitor the device throughout the race may complicate glucose management and introduce interruptions, especially in unfamiliar environmental conditions [43].

While insulin pumps and advanced hybrid closed-loop systems support flexible insulin dose adjustments in runners with type 1 diabetes (T1DM), they are not essential for participation in long-distance events such as marathons. In our group, none of the participants used insulin pumps, yet all completed the marathon safely—comparable to outcomes reported in other studies involving individuals with T1DM [16,24,42].

### 4.5. Study Limitations

Our study has limitations. The small sample size restricts the generalizability of the findings. Future studies should include more participants by collaborating with diabetes organizations and social media groups, recruiting runners from diverse cities and countries. Multicenter studies could help to validate and expand upon these results across broader populations.

Another limitation is the lack of a control group. Comparing outcomes with non-diabetic runners or a larger cohort of individuals with type 1 diabetes can enhance the interpretability of the results.

Although two CGM models were used, device-specific variations may have contributed to inconsistencies in glucose measurements. Moreover, factors like diet and exercise intensity were self-reported, potentially introducing bias. Due to the lack of time at the checkpoints, and the many feeding points along the way, assessing the amount of carbohydrates consumed was not precise. Future research with a larger sample and standardized conditions across devices is recommended to address these limitations.

## 5. Conclusions

This study demonstrates that individuals with T1DM can complete marathons with appropriate glycemic management strategies. Pre-race preparation, including maintaining a good time in range (TIR) and using continuous glucose monitoring (CGM), alongside self-monitoring blood glucose, can help to mitigate the risks of hypoglycemia and hyperglycemia during prolonged aerobic exercise. However, the occurrence of symptomatic hypoglycemia in several participants highlights the continued challenges of glucose regulation in endurance sports, even with advanced monitoring technologies.

The findings suggest that a combination of CGM, SMBG, and strategic carbohydrate intake is essential for optimizing glycemic control during marathons. Further research with larger cohorts is needed to refine strategies for glucose management in athletes with T1DM, enabling their safer participation in endurance events like marathons.

## Figures and Tables

**Table 1:** Recommendations for runners and medical team during the marathon.

Blood Glucose Level [mg/dL]	Recommendations	Comment
140–200	Target glycemia before start	
<150	15–30 g of carbohydrates	Real-time glucose monitoring by a medical team using a cloud-based platform
150–249	No intervention	
250–299 with ketone level ≥ 0.6 mmol/L (3.49 mg/dL)	Insulin correction factor with a 50% reduction in the correction dose at checkpoints In case of ketone level ≥ 1.5 mmol/L (8.71 mg/dL) → stop running	
≥300		

**Table 2:** General characteristics of type 1 diabetes marathon runners.

Parameter	Participant 1	Participant 2	Participant 3	Participant 4	Participant 5	All Participants (n = 5)
Age [years]	32	34	48	44	55	44 (34–48)
Diabetes duration [years]	6	10	1	14	42	10 (6–14)
Diabetes complications	-	-	-	-	Non-proliferative retinopathy	1/5
Time spent on sports per week [h]	12	6	12	3.5	4	6 (4–12)
Running time [years]	8	8	13	5	11	8 (8–11)
Height (m)	1.8	1.83	1.72	1.89	1.77	1.8 (1.8–1.8)
Body mass (kg)	73	84	65.1	78	73	73 (73–78)
BMI [kg/m^2^]	22.5	25.1	22.0	21.8	23.3	22.5 (22–23.3)
HbA1c [%]	5.8	6.9	5.3	5.6	6.9	5.8 (5.6–6.9)
TIR [%]	70.0	60.0	100.0	91.0	67.0	70 (65–90)
TAR [%]	17.0	37.0	0	8.0	32.0	20 (4–34.5)
TBR [%]	13.0	3.0	0	1.0	1.0	1 (0.5–8)
Average glycemia	118.0	158.0	107.0	130.0	163.0	130 (118–158)
CV [%]	44.0	39.5	12.5	27.8	34.6	34.6 (27.8–39.5)
Basal insulin per day [U]	13.0	24.0	0.0	22.0	7.0	13 (7–22)
Prandial insulin per day [U]	30.0	24.0	6.0	16.5	12.0	16.5 (12–24)
Marathon time [min]	188.9	239.3	268.6	299.9	218.0	239.3 (218.0–268.6)

HbA1c—glycated hemoglobin. TIR—time in range. CV—coefficient of variation in glycemia. BMI—body mass index.

## Data Availability

The data that support the findings of this study are available on reasonable request from the corresponding author.

## Abbreviations

The following abbreviations are used in this manuscript:

ADA	American Diabetes Association
BMI	Body Mass Index
CGM	Continuous Glucose Monitoring
CPET	Cardiopulmonary Exercise Testing
CSII	Continuous Subcutaneous Insulin Infusion
DG6	Dexcom G6
EASD	European Association for the Study of Diabetes
FSL2	FreeStyle Libre 2
HbA1c	Hemoglobin A1c
ISPAD	International Society for Pediatric and Adolescent Diabetes
MDI	Multiple Daily Injections
SMBG	Self-Monitoring of Blood Glucose
T1DM	Type 1 Diabetes Mellitus
TIR	Time in Range
VO_2_max	Maximal Oxygen Uptake
